# Prediction and Performance of BDS Satellite Clock Bias Based on CNN-LSTM-Attention Model

**DOI:** 10.3390/s26020422

**Published:** 2026-01-08

**Authors:** Junwei Ma, Jun Tang, Hanyang Teng, Xuequn Wu

**Affiliations:** Faculty of Land Resources Engineering, Kunming University of Science and Technology, Kunming 650093, China; junweima@stu.kust.edu.cn (J.M.); tenghanyang@stu.kust.edu.cn (H.T.); xuequnwu@kust.edu.cn (X.W.)

**Keywords:** SCB, BDS satellite, CNN, LSTM, attention mechanism, dynamic PPP

## Abstract

Satellite Clock Bias (SCB) is a major source of error in Precise Point Positioning (PPP). The real-time service products from the International GNSS Service (IGS) are susceptible to network interruptions. Such disruptions can compromise product availability and, consequently, degrade positioning accuracy. We introduce the CNN-LSTM-Attention model to address this challenge. The model enhances a Long Short-Term Memory (LSTM) network by integrating Convolutional Neural Networks (CNNs) and an Attention mechanism. The proposed model can efficiently extract data features and balance the weight allocation in the Attention mechanism, thereby improving both the accuracy and stability of predictions. Across various forecasting horizons (1, 2, 4, and 6 h), the CNN-LSTM-Attention model demonstrates prediction accuracy improvements of (76.95%, 66.84%, 65.92%, 84.33%, and 43.87%), (72.59%, 65.61%, 74.60%, 82.98%, and 51.13%), (70.45%, 68.52%, 81.63%, 88.44%, and 60.49%), and (70.26%, 70.51%, 84.28%, 93.66%, and 66.76%), respectively, across the five benchmark models: Linear Polynomial (LP), Quadratic Polynomial (QP), Autoregressive Integrated Moving Average (ARIMA), Backpropagation Neural Network (BP), and LSTM models. Furthermore, in dynamic PPP experiments utilizing IGS tracking stations, the model predictions achieve positioning accuracy comparable to that of post-processed products. This proves that the proposed model demonstrates superior accuracy and stability for predicting SCB, while also satisfying the demands of positioning applications.

## 1. Introduction

Real-time Precise Point Positioning (PPP) technology is now used in various fields, including surveying, geographic information systems, and unmanned vehicles. The growing demand for high-accuracy localization underscores the necessity of a high-precision time system [1,2]. The PPP technique hinges on continuous and precise satellite orbits and clocks. Since April 2013, the International GNSS Service (IGS) has offered Real-Time Service (RTS), providing global users with satellite orbits, clock differentials, and other products for both real-time and post-processing applications [3,4,5]. The Final Precise Clock Products provided by IGS have a high accuracy of 75 ps but are published as post-processing data with a delay of about two weeks, making them unusable for real-time applications. The Ultra-Rapid Clock Products are for real-time service, but its accuracy is limited to 3 ns, insufficient for high-precision positioning [6]. At the same time, limitations in network connectivity and localization areas can cause interruptions in the real-time RTS data stream, which in turn affects the accuracy of localization [7]. Thus, forecasting Satellite Clock Bias (SCB) with high-precision post-processing data has been becoming a new method to derive the correction with feasible precision for real-time positioning [8,9,10].

The current commonly used models for SCB prediction include the Quadratic Polynomial (QP) model [11], Gray Model (GM) [12,13], Spectral Analysis (SA) [14], Kalman Filter (KF) [15], and Autoregressive Integrated Moving Average (ARIMA) [16]. These models have greatly improved SCB forecasting, while each model has specific advantages and limitations because of the nonlinear and stochastic nature of SCB. The QP model is computationally simple and delivers superior accuracy for short-term predictions, but the stability and accuracy decrease with increasing forecasting period. The GM model assumes a smooth, exponential trend in the underlying function; otherwise, its predictive performance deteriorates significantly. The KF model predicts SCB by incorporating the motion properties of atomic clocks and stochastic a priori knowledge. The ARIMA model predicts most accurately when its p and q parameters are chosen appropriately. The SA model can achieve higher prediction accuracy, but a very long and stable time series are required. Satellite atomic clocks are time-varying complex and influenced by multiple factors, leading to complexity in SCB estimation. This is precisely where machine learning techniques specialize because they can generate predictions by learning patterns and relationships from historical data in combination with mathematical algorithms [17]. Wang et al. [18] used a Radial Basis Function (RBF) model to predict SCB of GPS satellites and achieved notable prediction accuracy. Similarly, Wang et al., [19] proposed a Wavelet Neural Network (WNN)-based SCB prediction model, which shows improved prediction accuracy compared to traditional QP and GM based models. However, the RBF method lacks a robust theoretical basis to select sample parameters, and the determination of the WNN structure remains challenging.

Among the many machine learning models, Recurrent Neural Networks (RNNs) are another widely used machine learning method, which utilizes the characteristics of network structure to search for sequence correlation by directionally connecting nodes into a loop. However, RNNs are prone to extreme nonlinear behavior due to multiple propagation during parameter updating. Subsequently, researchers introduced the “gate” mechanism and proposed the Long Short-Term Memory Neural Network (LSTM), which effectively solved the parameter problem of RNNs and demonstrated improved prediction accuracy. Currently, the forecasting of SCBs using LSTM networks has also achieved good results, and the LSTM forecasting algorithms [20,21] have fitted the satellite clock difference data to make predictions, which have achieved good prediction results. The QP-LSTM model [22] integrates a conventional model to enhance its performance in predicting a single data type. The LSTM-Attention model [23] leverages a self-attention mechanism to effectively balance global attention with local feature capture, thereby enhancing both prediction accuracy and stability.

Building upon the advancements of LSTM and its variants, existing models have significantly improved the prediction of SCB. However, two key aspects warrant further exploration to enhance performance. First, from an architectural perspective, most approaches focus on optimizing a single component, leaving the potential of a coherent, synergistic integration of these complementary strengths under-explored. Second, from a data utilization perspective, the prevailing single-satellite paradigm predicts each clock bias independently, potentially overlooking valuable inter-satellite correlations and constellation-level systematic behaviors that could serve as informative auxiliary features. To address these gaps, this study proposes a novel hybrid CNN-LSTM-Attention architecture that not only integrates the strengths of these components into a unified framework but also innovatively employs a multivariate input strategy to leverage data from other satellites as contextual features. First, the Convolutional Neural Network (CNN) extracts effective features in satellite clock offsets through the operation of convolutional kernels to obtain an accurate representation of the main features. Then LSTM simulates the nonlinear trend of satellite clock deviation and extracts the timing change information. Finally, the attention mechanism applies adaptive weights to the LSTM outputs to generate the final SCB prediction. Among them, we attempted a multivariate input prediction approach. In the CNN-LSTM-Attention model, the SCB of a single satellite is taken as the primary prediction target, while data from other satellites are incorporated as feature inputs into the prediction model. This process is sequentially carried out to complete the prediction tasks for all BDS satellites. Finally, we conducted simulated real-time PPP experiments using the predicted satellite clock error data. A rigorous quality assessment of IGS tracking station data confirms the practical utility and robustness of the proposed model.

## 2. Data Sources

We evaluated the proposed model and algorithm for clock difference forecasting. IGS was utilized to provide after-the-fact precision satellite clock difference data, which was downloaded from https://cddis.nasa.gov/archive/gnss/products (accessed on 25 June 2025). The data are selected from DOY 11, 2024 with a sampling interval of 30 s. This study focuses on the modeling, forecasting, and analysis of post-processed high-precision satellite clock offsets from the BDS system. The BDS constellation includes satellites in three types of orbits: geostationary orbit (GEO), inclined geosynchronous orbit (IGSO), and medium-Earth orbit (MEO), equipped with either rubidium or hydrogen atomic frequency standards. For this analysis, we selected only satellites with complete SCB data, defined as the uninterrupted 24 h series sampled at 30 s intervals (containing 2880 data points). Table 1 presents their designations, orbit types, and onboard clock types.

**Figure 1:**
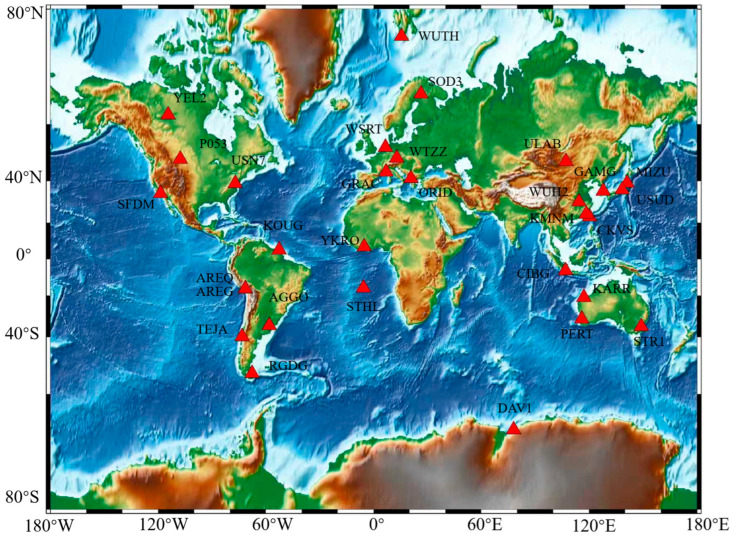
Location information of 30 IGS observation stations from the WUH Data Center.

The model’s prediction performance was evaluated through real-time kinematic PPP experiments. For this purpose, observational data from 30 globally distributed tracking stations of the International GNSS Service were utilized. The specific coordinates of these stations are provided in Table 2, and their geographical distribution is illustrated in Figure 1. The IGS stations are shown as red triangles in Figure 1. Among them, the IGS station is sourced from the WHU data center, and the station data is sourced from ftp://igs.gnsswhu.cn/pub/gps/data/daily/ (accessed on 15 September 2025).

## 3. Model and Methodology

### 3.1. CNN Principle

CNNs are feedforward neural networks that utilize convolutional computations and deep architectures. Initially developed for image classification, object detection, image segmentation, and other visual tasks, CNN have achieved remarkable success in the field of computer vision. Moreover, they demonstrate significant potential across multiple domains, spanning the fields of speech recognition, natural language processing, and time series forecasting [24]. The core of convolutional computation is the operation of sliding kernels across input data to identify locally relevant features. These local features can be hierarchically combined to form higher-level abstract representations, thereby enabling effective analysis and understanding of the input data. In this study, the CNN component is employed to extract locally relevant features from the SCB data. This is achieved through the sliding operation of convolutional kernels across the input sequence, which hierarchically identifies and combines patterns to form effective feature representations for subsequent processing. The convolutional operation in the CNN model is implemented by sliding convolutional kernels over the input sequence. This process effectively captures local dependencies and spatial patterns within the data, and its computation can be formally described as follows:

(1) $$h_{k}=\sigma (W_{k}*x+b_{k})$$

(2) $$W_{k}*x_{i,y}=\sum_{m=0}^{\alpha -1}\sum_{n=0}^{\beta -1}w_{m,n}\times x_{i+m,y+n}$$

where *x* is the input data; $h_{k}$ denotes the output data; σ denotes the sigmoid activation function; $W_{k}$ and $b_{k}$ are the weight coefficients and bias functions, respectively; *k* denotes the number of convolutional kernels of the network; ∗ denotes the discrete convolution operation; α and β denote the size parameters of the convolution kernel; $w_{m,n}$ is a matrix of weight functions and *m* and *n* are the rows and columns of the matrix.

### 3.2. LSTM Principle

**Figure 2:**
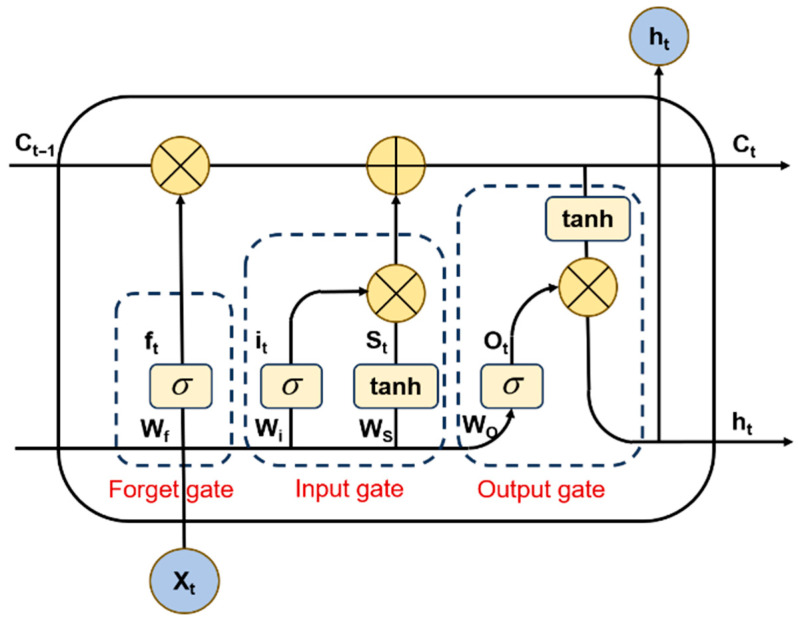
LSTM neural network structure diagram.

The LSTM network is a specialized type of RNN designed to model temporal sequences and capture long-range dependencies. Its core innovation lies in the introduction of a gated mechanism, which overcomes the vanishing and exploding gradient problems inherent in standard RNNs when processing long sequences [25,26]. This mechanism enables LSTM to effectively control and retain information over extended time periods, making it particularly suited for time series analysis and prediction tasks. As illustrated in Figure 2, this structure consists of a cell state and three regulatory gates (input, forget, and output gates), which collectively manage the flow of information. In the LSTM unit, the information flow is regulated by three gates: the forget gate, the input gate, and the output gate. The detailed computational process is as follows:

(3) $$f_{t}=\sigma (W_{f}\left[h_{t-1},x_{t}\right]+b_{f})$$

(4) $$i_{t}=\sigma (W_{i}\left[h_{t-1},x_{t}\right]+b_{i})$$

(5) $$o_{t}=\sigma (W_{o}\left[h_{t-1},x_{t}\right]+b_{o})$$

(6) $$\tilde{C_{t}}=\tanh(W_{C}\left[h_{t-1},x_{t}\right]+b_{C})$$

(7) $$C_{t}=f_{t}\cdot C_{t-1}+i_{t}\cdot \tilde{C_{t}}$$

(8) $$h_{t}=o_{t}\cdot \tanh(C_{t})$$

where σ denotes the sigmoid activation function; tanh is the hyperbolic tangent activation function; $f_{t}$, $i_{t}$ and $o_{t}$ are the forget gate, input gate and output gate, respectively; *W* and *b* are the weight coefficients and bias function, respectively; $x_{t}$, $h_{t}$, $\tilde{C_{t}}$ and $C_{t}$ are the input information, output information, candidate cell state and updated cell state at time *t*, respectively.

Each LSTM cell contains three gates: forget, input, and output. The forget gate regulates the retention of prior information; the input gate regulates the integration of new data; and the output gate regulates the exposure of the cell state. Collectively, these gates enable long-term memory retention, allowing the model to handle long sequences more effectively.

### 3.3. CNN-LSTM-Attention Model

The attention mechanism is inspired by human cognition, which selectively focuses on salient information while filtering out irrelevant stimuli from complex, high-volume inputs. In machine learning, this is implemented as a computational resource allocation strategy that amplifies key features and suppresses less useful ones, effectively learning to assign varying weights to different parts of the input [27].

**Figure 3:**
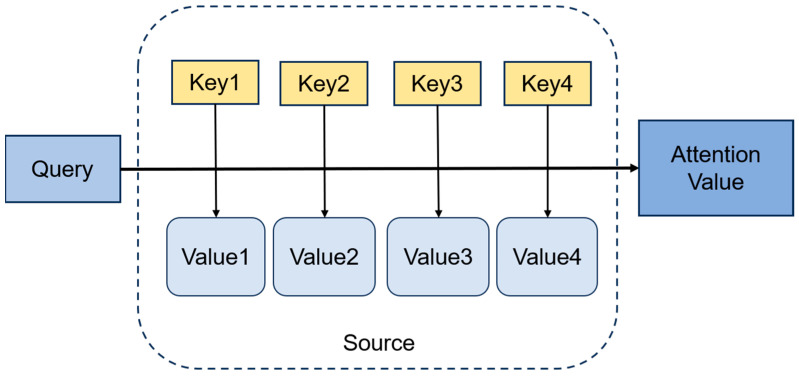
Attention Mechanism Flowchart.

The structure of the attention mechanism, depicted in Figure 3, is built upon three primary vectors, namely the Query, Key, and Value. The query vector refers to the range of the query and represents the feature vector of subjective awareness. The Query vector serves as the primary reference for attention. It is generated from either the input sequence or its previous hidden state. The key vector is a vector of salient feature information about the object. It represents the feature vector of each input element, against which the Query is matched to compute the attention weights. The value vector is the feature vector representing the object itself, which usually appears in pairs with the key vector. The Value vector represents the actual output for each input element, and the final attention output is computed as a weighted sum of these values based on the attention weights. These three vectors cooperate with each other to realize the calculation process of the attention mechanism. Through the attention convergence of Query and Key to realize the attention weight allocation to Value, to generate the final output results.

**Figure 4:**
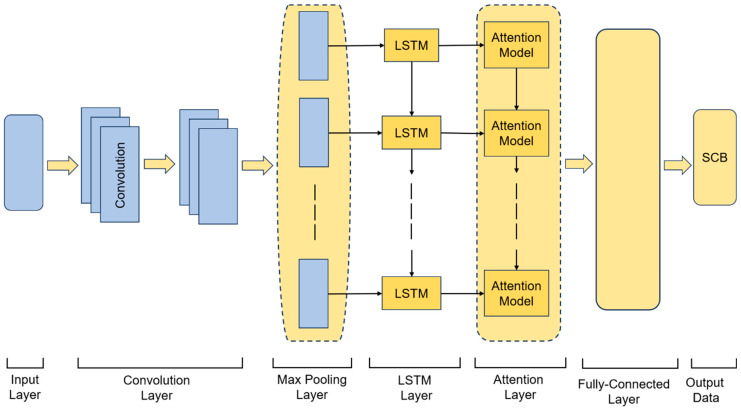
Architecture of the CNN-LSTM-Attention Model.

We propose a CNN-LSTM-Attention model for SCB prediction, as illustrated in Figure 4. The architecture sequentially integrates an input layer, a CNN module, an LSTM layer, an attention mechanism, and a fully connected output layer. The CNN module, comprising two 1D convolutional layers followed by max-pooling operations, extracts local features and short-term patterns from the input SCB time series while reducing dimensionality and enhancing robustness. The extracted features are then fed into the LSTM layer, which captures long-term trends and complex nonlinear dependencies in the data through its gated cell structure, outputting a sequence of hidden states that encode temporal information.

Subsequently, an attention mechanism assigns adaptive weights to the LSTM hidden states via a fully connected layer and a SoftMax function, quantifying the contribution of each time step to the final prediction. A context vector is generated as the weighted sum of these states, emphasizing the most relevant historical information. This integrated design ensures synergistic cooperation: the CNN focuses on local feature extraction, the LSTM models long-term temporal dependencies, and the attention mechanism dynamically highlights critical time steps. The context vector is finally passed through a fully connected layer to produce the SCB prediction, collectively improving both the accuracy and stability of the forecasting results.

**Figure 5:**
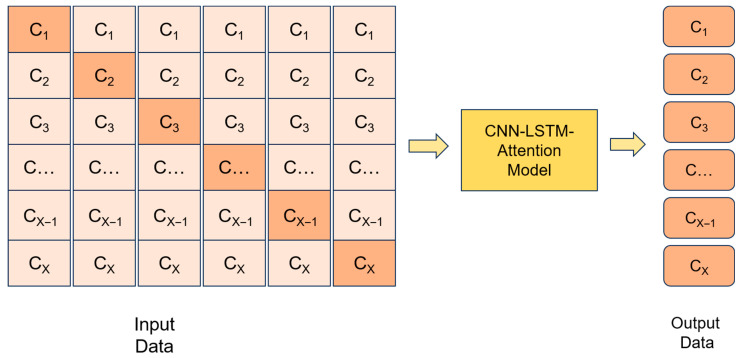
Predictive Modeling Methodology.

Subsequently, researchers employed the Pearson correlation analysis method to investigate the inter-satellite correlations among different BeiDou satellites [28] and proposed a parallel predictive multi-satellite joint clock error forecast model. By integrating CNN with LSTM networks, this model utilizes an inter-satellite correlation-based multivariate prediction approach to simultaneously forecast clock errors for multiple satellites, thereby enhancing prediction performance. This study explores a multivariate forecasting approach, as illustrated in Figure 5, where the SCB of a single satellite serves as the primary prediction target, while data from other satellites are incorporated as input features into the model. The CNN-LSTM-Attention model is utilized to sequentially complete the SCB prediction tasks for all BDS satellites.

In this context, CX denotes the SCB values corresponding to BDS satellites with different PRN numbers. The SCB data from all satellites within the entire constellation will be utilized as input for the model. The system will then proceed to sequentially generate SCB predictions for each BDS satellite.

### 3.4. Data Pre-Processing and Parameter Settings

SCB constitutes a univariate time series characterized by a limited number of sampling points and typically exhibits an underlying linear trend for a given satellite. The generation of SCB is influenced by a combination of gravitational, velocity, and relativistic effects, making it difficult to derive accurate features directly from these physical influences. In practice, ground control systems worldwide determine the SCB by comparing the satellite’s clock time with the standard GPS time using historical tracking data. Forecasts are then generated either by modeling the specific clock’s characteristics or by applying extrapolation techniques to the analyzed deviation trends.

When using deep learning network models for SCB prediction, they are affected by raw data series with linear trends, resulting in poor extraction of nonlinear data features. Therefore, we remove the constant term from the SCB data sequence by applying first-order differencing according to Equations (11) and (12). This centers the differenced series around zero, thereby making its underlying variation and trend more pronounced for subsequent analysis.

First, the Median Absolute Deviation (MAD) method [29] is employed to detect and process coarse differences in the raw satellite clock difference sequence, where the bit number equation is:

(9) $$T_{MAD}=Median\left[\frac{\left|y_{i}-mid\right|}{0.6745}\right]$$

where *mid* means the value in the middle of the clock difference sequence. If $|y_{i}|>mid+x\cdot T_{MAD}$ (*x* ∈ (1, 5), and usually *x* = 3), it is considered to be an outlier and replaced by the value calculated by segmented linear interpolation. The processed SCB data are arranged into n-dimensional sequences:

(10) $$X=\{x_{1},x_{2},x_{3},\cdot \cdot \cdot ,x_{t}\}$$

where *x* denotes the original SCB data sequence from the first data point (i.e., the first observation in the series) to the *t*-th data point. The data sequence is then differenced once to obtain a first-order differenced data sequence.

(11) $$\Delta x_{i}=x_{i+1}-x_{i}$$

(12) $$X_{\Delta }=\{\Delta x_{1},\Delta x_{2},\Delta x_{3},\cdot \cdot \cdot ,\Delta x_{t-1}\}$$

However, for each satellite, the first-order differenced SCB series values have a different range of numerical magnitudes. To mitigate scale discrepancies after first-order differencing and prevent any single variable from dominating the analysis, the data were normalized to the [0, 1] interval using min-max scaling. This process enhances model stability and generalization. The normalization is defined as:

(13) $$\tilde{\Delta x_{i}}=\frac{\Delta x_{i}-X_{\Delta \max}}{X_{\Delta \max}-X_{\Delta \min}}$$

(14) $$\tilde{X_{\Delta }}=\{\tilde{\Delta x_{1}},\tilde{\Delta x_{2}},\tilde{\Delta x_{3}},\cdot \cdot \cdot ,\tilde{\Delta x_{t-1}}\}$$

where $X_{\Delta \max}$ and $X_{\Delta \min}$ are the maximum and minimum values in the first-order differenced SCB data sequence, respectively, and $\Delta x_{i}$ denotes the data of the *i*-th calendar element. Finally, we will group the normalized data using a sliding window to reduce the computational complexity. A window size of m is defined, segmenting the normalized data into *t*-*m*-1 input-output sequences for supervised training.

(15) $$\begin{array}{l} \left\{\tilde{\Delta x_{1}},\tilde{\Delta x_{2}},\tilde{\Delta x_{3}},\cdot \cdot \cdot ,\tilde{\Delta x_{m},}\tilde{\Delta x_{m+1}}\right\}\\ \left\{\tilde{\Delta x_{2}},\tilde{\Delta x_{3}},\tilde{\Delta x_{4}},\cdot \cdot \cdot ,\tilde{\Delta x_{m+1},}\tilde{\Delta x_{m+2}}\right\}\\ \cdot \cdot \cdot \\ \left\{\tilde{\Delta x_{t-m-1}},\tilde{\Delta x_{t-m}},\tilde{\Delta x_{t-m+1}},\cdot \cdot \cdot ,\tilde{\Delta x_{t-2},}\tilde{\Delta x_{t-1}}\right\} \end{array}$$

**Figure 6:**
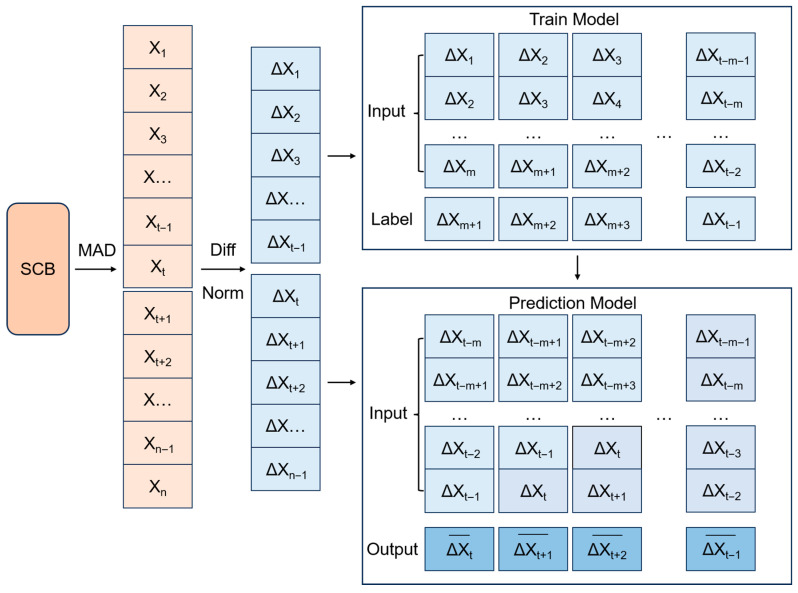
Data Preprocessing Flowchart.

For each small sample dataset, we use the first m data as input data to the model, and the last point as the label of the predicted value, and input all the arrays into the model sequentially for SCB prediction. Figure 6 illustrates the complete data processing workflow, from the input of the raw SCB sequence through model training to final prediction.

In the training phase, we predict the *m*+1th data point using the previously known m data points. And in the prediction phase, the first prediction value will be predicted based on the previous m data points. Subsequently, in the second prediction step, the window is advanced by one time step: the earliest data point is removed, the latest observation is incorporated, and the prediction for the *m*+2nd point is generated based on the updated sequence of the most recent m points. Finally, a sliding window of m data lengths will be utilized sequentially to complete our prediction task.

Finally, upon completion of the aforementioned prediction tasks, we obtained the preliminary prediction outputs. To further restore their practical significance, the data must undergo post-processing procedures, primarily involving denormalization and linear transformation. Denormalization remaps the standardized data output by the model back to the original value range to recover its actual physical dimensions, while linear transformation ensures that the predicted results reside in the same space as the true values. Through these steps, the final directly applicable SCB prediction results are obtained. See Table 3 for detailed experimental parameters.

This paper presents a hybrid CNN-LSTM-Attention model implemented on the TensorFlow framework; its parameter configurations are detailed in the accompanying table. The proposed architecture processes time series data through sequential layers: A 1D CNN with 64 filters (kernel size = 5) for local feature extraction, followed by an LSTM layer (64 hidden units) to capture long-term dependencies. The model was trained for 200 epochs using mini-batches of 64, with Mean Squared Error (MSE) as the loss function and the Adam optimizer [30] for stable convergence. The network accepts an input dimension of 64 and produces a single output, representing the predicted satellite clock bias. This integrated design effectively synergizes convolutional, recurrent, and attention mechanisms to achieve high-precision forecasting.

## 4. Experimentation and Analysis

After preprocessing, the short-term forecasting experiments are performed using the prediction model developed in this study as well as other conventional models. The known precision SCB for the forecast period serves as the ground truth to evaluate each model’s prediction accuracy. Error, root mean square error (RMSE) and Mean error are used as the indexes of clock difference accuracy, and their calculation formulas are as follows:

(16) $$Error={\hat{y}}_{SCB_{i}}-y_{SCB_{i}}$$

(17) $$RMSE=\sqrt{\frac{1}{m}\sum_{i=1}^{m}{\left({\hat{y}}_{SCB_{i}}-y_{SCB_{i}}\right)}^{2}}$$

(18) $$Mean=\frac{1}{m}\sum_{i=1}^{m}\left({\hat{y}}_{SCB_{i}}-y_{SCB_{i}}\right)$$

(19) Improvement-Rate=Mbase−MnewMbase×100%

where *m* is the number of data epochs, ${\hat{y}}_{SCB_{i}}$ represents the predicted SCB at the *i*-th epoch, $y_{SCB_{i}}$ denotes the measurements at the *i*-th epoch. For multiple satellites, the average RMSE served as the clock error accuracy metric. *M* denotes the evaluation metric. For error-based metrics, the formula is adjusted so that a positive value indicates performance enhancement.

### 4.1. Performance Assessment of Forecasting Models

The forecasting performance of the CNN-LSTM-Attention model was evaluated by benchmarking it against five established models: LP, QP, ARIMA, BP, and LSTM. In this study, we utilize the post-processed precise SCB data for DOY 11, 2024, available from the IGS Wuhan University Data Center (WHU). The first 18 h of SCB data from that day were used for model training and fitting. Subsequently, the trained model was applied to perform one-step-ahead predictions for four different forecast horizons (1 h, 2 h, 4 h, and 6 h). Specifically, each prediction task was accomplished through a single sliding-window prediction step, rather than employing an iterative rolling or recursive prediction process. We selected the known precise SCB from the forecast period as the ground truth. Against this reference, the overall mean prediction accuracy across all satellites was computed. As summarized in Table 4 and the accompanying figure, the prediction performance across models is presented through statistical data and accuracy metrics, respectively.

The determination of the hyperparameters for each model is described as follows: or the ARIMA model, the order parameters (p, d, q) were automatically determined through a grid search within a pre-defined range, with p ranging from 0 to 2, d from 0 to 1, and q from 0 to 2., and the optimal parameter combination was selected based on the minimized Bayesian Information Criterion (BIC), aiming to achieve a balance between model fit and simplicity. The LSTM model adopts a single-layer LSTM architecture with 500 hidden units, incorporating a Dropout layer (rate = 0.2) and a ReLU activation function to mitigate overfitting. The model was trained using the Adam optimizer, with a maximum of 600 epochs, an initial learning rate of 0.005, and a piecewise learning rate decay strategy to enhance training performance. The BP neural network features a three-layer feedforward structure, consisting of 15 input nodes, 5 hidden nodes, and 1 output node. The Particle Swarm Optimization algorithm was employed to initialize the network’s weights and thresholds, with a population size of 5, 30 iteration steps, and an adaptive mutation mechanism to improve global search capability. The network was trained for a maximum of 2000 epochs with a learning rate of 0.001, and data normalization and denormalization were applied to enhance training stability and prediction accuracy.

**Figure 7:**
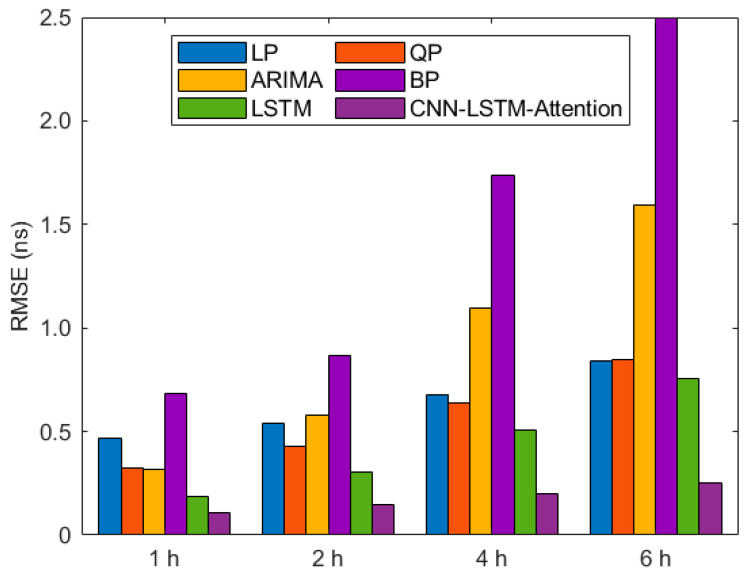
The RMSE for tasks with different forecast time periods.

The overall superiority of the CNN-LSTM-Attention model is evidenced by the results in Table 4 and Figure 7 Compared with LP, QP, ARIMA, BP and LSTM models, the prediction accuracy shows a percentage improvement rate of 76.95%, 66.84%, 65.92%, 84.33%, and 43.87% in the 1 h prediction task; 72.59%, 65.61%, 74.60%, 82.98%, and 51.13% in the 2 h prediction task; 70.45%, 68.52%, 81.63%, 88.44%, and 60.49% in the 4 h prediction task; 70.26%, 70.51%, 84.28%, 93.66%, and 66.76% in the 6 h prediction task.

Notably, the model’s superiority over traditional forecasts remains significant with extending prediction horizons. In contrast, its improvement compared to the BP neural network and LSTM models continues to expand with longer forecast durations. These results indicate that the model excels in short-term forecasting, while also demonstrating more stable advantages over traditional LSTM methods in long-term prediction tasks. This suggests that the model possesses strong adaptability and generalizability across various time scales in satellite clock bias prediction.

**Figure 8:**
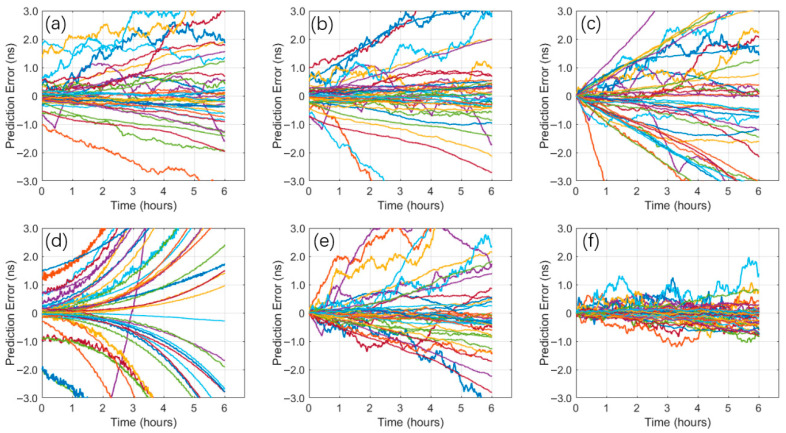
Prediction residuals from different models: LP (**a**), QP (**b**), ARIMA (**c**), BP (**d**), LSTM (**e**), and CNN-LSTM-Attention (**f**) (Each curve represents the predicted SCB error of a different satellite, with distinct colors used to differentiate between satellites).

Figure 8 illustrates the trend of forecast errors in the prediction task, with curves in different colors representing different BDS satellites. As shown in Figure 8, forecast errors accumulate across all six models as the prediction time lengthens. The CNN-LSTM-Attention model demonstrates superior error control capability throughout the entire prediction period. Its prediction error sequence is noticeably more convergent, with a significantly smaller fluctuation range compared to the LP, QP, ARIMA, BP, and LSTM models. Especially under longer prediction time horizons, the model still maintains a lower error level without exhibiting obvious divergence, reflecting its excellent stability and generalization performance. In contrast, the other models show gradually increasing errors after the medium to long term, and their error distributions remain more dispersed than that of the CNN-LSTM-Attention model. The results indicate that the CNN-LSTM-Attention model can more effectively capture complex patterns in clock bias sequences and exhibits consistent prediction performance across different time scales.

**Figure 9:**
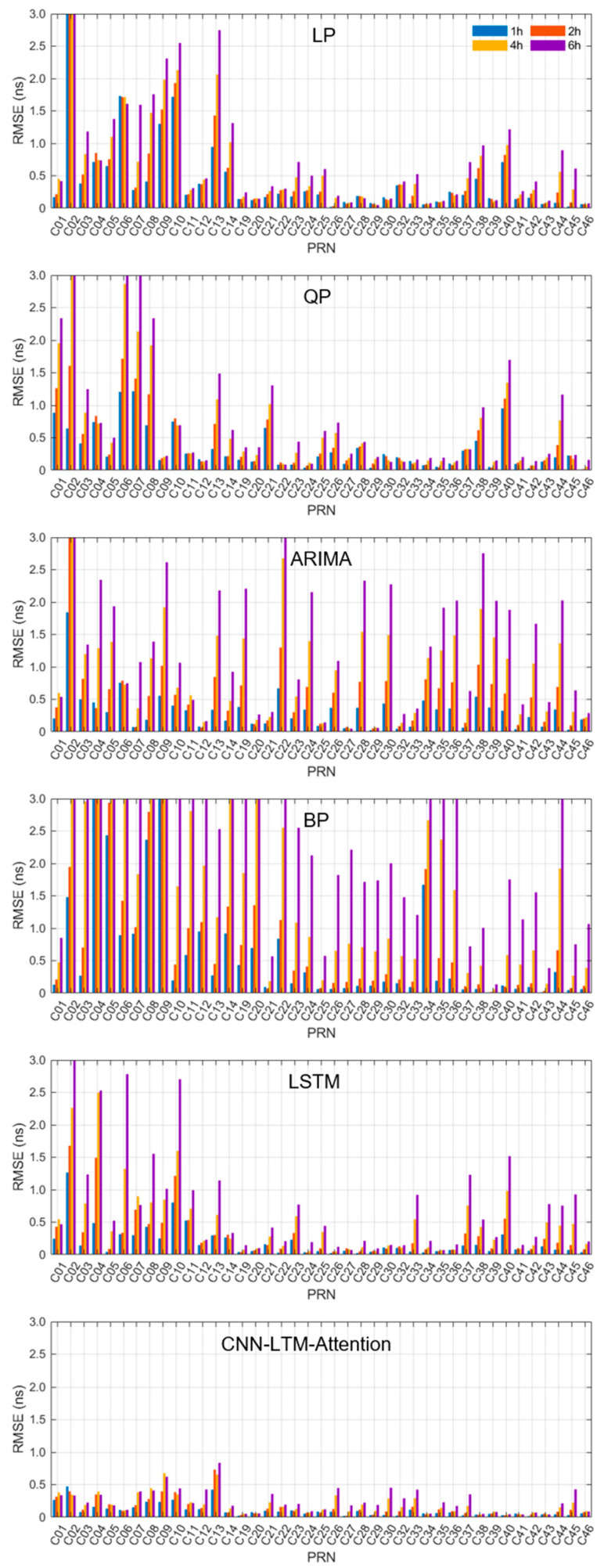
The average prediction accuracy of each satellite in different models obtained based on different prediction tasks (shown from top to bottom).

As shown in Figure 9 and Table 5, the CNN-LSTM-Attention model achieves the best accuracy for the majority of satellites. The overall RMSE values across all satellites exhibit minimal fluctuation, demonstrating its high stability and consistent performance. These results indicate that the model not only improves prediction accuracy but also exhibits strong generalization capability across different satellites. Compared to other benchmark models, the proposed architecture shows a superior ability to capture complex patterns in satellite clock bias data, thereby enhancing its reliability across all forecasting horizons. Given these advantages, the model is ideal for a range of high-precision applications, particularly real-time positioning and timing systems.

### 4.2. PPP Experiment

The predicted SCB products were employed for PPP calculation to evaluate the model’s performance. Specifically, kinematic PPP data processing was performed for the selected IGS tracking stations mentioned above. The positioning results were then compared with those obtained using the post-processed SCB products available from the WHU to verify the predictive performance. Here, data from post-processed products covering the experimental prediction interval were selected for simultaneous kinematic PPP processing. In the experiment, we utilized the RTPPP software developed by GFZ to conduct real-time PPP tests. The processing mode was set to “kinematic for positioning verification”. Antenna phase center corrections were applied using the “IGS 20.atx” model, with an elevation cut-off angle of 7°. For the antenna phase center corrections in this study, we utilized the product data disseminated by the IGS, available at: https://files.igs.org/pub/station/general (accessed on 15 September 2025). Tropospheric delay was modeled using ZHD with the Saastamoinen model, while ionospheric delay was addressed by eliminating first-order effects via dual-frequency GNSS signals. For satellite orbits, WUH precision satellite orbits were adopted, and clock corrections were based on 6 h predicted SCB data from the aforementioned forecasting experiment. The calculated station coordinates in simulated real-time positioning mode were compared with the known static coordinates of the IGS stations. In this context, dynamic PPP continuously processes GNSS observations in real time, updating receiver coordinates epoch by epoch. This method simulates a dynamic or kinematic scenario in practical positioning applications, where the receiver may be in motion and its position changes over time. By employing predicted satellite clock and orbit products in this kinematic PPP framework, the experiment effectively evaluates the performance of the forecasting model under real-world, time-varying conditions, thereby providing a realistic assessment of the predictive SCB products’ capability in supporting high-precision real-time navigation and positioning.

Subsequently, we compared the average positioning errors between the predicted and product data in the N, E, and U directions. Table 6 summarizes the positioning results. The statistical analysis excluded PPP results from the first two hours. The kinematic PPP with BDS satellites achieves positioning accuracy comparable to that of post-processed SCB products in all directions. This result confirms both the effectiveness and practicality of the prediction model. The horizontal positioning accuracy (N and E) is notably higher, with most station errors within 10 cm, while the vertical (U) direction shows slightly larger discrepancies.

**Figure 10:**
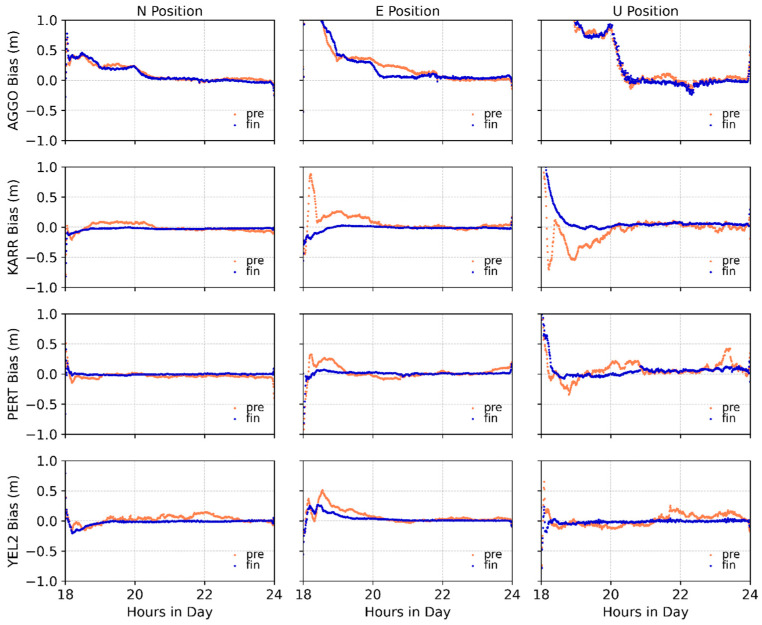
Comparative diagram of positioning errors.

Comparison between the positioning results derived from the predicted data and the post-processed SCB products reveals a strong consistency across the vast majority of stations, with both sets of results exhibiting positioning accuracy of the same order of magnitude. Although the post-processed precise products demonstrate superior accuracy at some stations (USN7, YEL2), the predicted data conversely achieve better performance at others (AGGO). The predicted SCB approach the accuracy of the post-processed products, demonstrating their capability to support real-time positioning requirements. Furthermore, as can be observed from the error sequences of stations AGGO, KARR, PERT, and YEL2 in Figure 10, the PPP results utilizing the predicted clock offsets are stable and reliable. The experimental results confirm that the employed clock offset prediction model can provide a reliable service for BDS real-time precise point positioning, holding significant practical value.

The positioning accuracy was subsequently assessed using the RMSE of the kinematic PPP solutions derived from the predicted data. As shown in Table 7, the results achieve decimeter-level accuracy across all stations, albeit with expectedly lower precision in the vertical component. Among them, some stations (such as KOUG and YKRO) exhibit relatively larger errors, while the majority of stations maintain high accuracy, confirming the effectiveness of the method for real-time positioning applications. The significant errors observed at stations KOUG and YKRO are primarily attributed to their coastal geographic locations near the equator. Such environments are highly susceptible to strong multipath effects, such as those caused by sea surface reflections. PPP solutions are particularly sensitive to this type of multipath interference, especially in coastal areas at low latitudes, where signal reflection and delay issues can further amplify positioning errors.

## 5. Conclusions

A CNN-LSTM-Attention model is proposed for SCB prediction. This approach enhances the forecasting accuracy of BDS satellite clock differences by leveraging all informative content in the data. Our experimental study reveals that:

(1) This paper describes the development of a hybrid model based on CNN, LSTM, and Attention mechanisms for predicting BDS SCB. In this architecture, local features extracted by the CNN layer are passed to the LSTM to model temporal dependencies. The Attention mechanism then enhances this process by adaptively weighting critical timesteps, collectively improving prediction accuracy and stability. Meanwhile, we sequentially complete the prediction tasks by leveraging the correlations among BDS satellites.
(2) Regarding both accuracy and stability, the CNN-LSTM-Attention model significantly outperforms all benchmark models (LP, QP, ARIMA, BP, LSTM). Furthermore, the model excels in short-term predictions and demonstrates remarkable stability in the long term, as the RMSE for most satellites stays below 1 ns.
(3) Finally, we conducted simulated real-time PPP experiments using predicted SCB data and post-processed SCB products over the same time period, performing kinematic PPP data processing for 30 selected globally distributed IGS tracking stations. Experimental results demonstrate comparable positioning accuracy between the predicted data and post-processed products in the N, E, and U directions, confirming its suitability for positioning applications.

Looking forward, to enhance the applicability of the model in continuous operational scenarios including multi-day or cross-day forecasting, we plan to incorporate a discontinuity correction mechanism. Daily boundary discontinuities, a recognized feature of continuous SCB products, will be addressed by applying a constant offset to align the clock bias series at day transitions. This approach is expected to eliminate prediction jumps and improve the consistency and robustness of long-term SCB forecasts in sustained positioning applications.

## Figures and Tables

**Table 1:** Information of satellites and atomic clocks in different BDS systems.

System	Model	Track	Clock	PRN
BDS	BDS-2	GEO	Rb	C01 C02 C03 C04 C05
		MEO		C11 C12 C14
		IGSO		C06 C07 C08 C09 C10 C13
	BDS-3	MEO	Rb	C19 C20 C21 C22 C23 C24 C32 C33 C36 C37 C41 C42
			PHM	C25 C26 C27 C28 C29 C30 C34 C35 C43 C44 C45 C46
		IGSO	PHM	C38 C39 C40

**Table 2:** Geographic coordinates of IGS tracking stations.

Stations	Latitude (°)	Longitude (°)	Stations	Latitude (°)	Longitude (°)
AGGO	−34.874	−58.140	RGDG	−53.786	−67.752
AREG	−16.465	−71.493	SFDM	34.460	−118.755
AREQ	−16.466	−71.493	SOD3	67.421	26.389
CIBG	−6.490	106.849	STHL	−15.943	−5.667
CKSV	22.999	120.220	STR1	−35.316	149.010
DAV1	−68.577	77.973	TEJA	−39.805	−73.253
GAMG	35.590	127.920	ULAB	47.865	107.052
GRAC	43.754	6.921	USN7	38.921	−77.066
KARR	−20.981	117.097	USUD	36.133	138.362
KMNM	24.464	118.389	WSRT	52.915	6.604
KOUG	5.098	−52.640	WTZZ	49.144	12.879
MIZU	39.135	141.133	WUH2	30.532	114.357
ORID	41.127	20.794	WUTH	77.003	15.539
P053	48.726	−107.725	YEL2	62.481	−114.481
PERT	−31.802	115.885	YKRO	6.871	−5.240

**Table 3:** Parameterization of CNN-LSTM-Attention models.

No.	Parameters	Value
1	Loss function	MSE
2	Optimizer	Adam (learning rate = 0.001)
3	Convolutional layer filters	64
4	Convolutional layer kernel size	5
5	Convolutional layer activation	ReLU
6	LSTM Hidden Layer Size	64
7	Attention layer activation	Sigmoid
8	Batch size	64
9	Training epoch	200
10	Input dimension size	64
11	Output dimension size	1
12	Train/Test split ratio	75%/25%
13	Data normalization	Min-Max scaling [0, 1]

**Table 4:** Average prediction accuracy RMSE for different models.

Model	1 h	2 h	4 h	6 h
LP	0.465	0.537	0.680	0.841
QP	0.323	0.428	0.638	0.849
ARIMA	0.314	0.580	1.095	1.592
BP	0.684	0.865	1.738	3.945
LSTM	0.189	0.301	0.509	0.753
CNN-LSTM-Attention	0.107	0.147	0.201	0.250

**Table 5:** Comparison of statistical results for the six models across different orbital types.

Prediction Task	Model	BDS-2			BDS-3		
	Track	GEO	MEO	IGSO	MEO		IGSO
	Clock	Rb			Rb	PHM	PHM
1 h	LP	1.357	0.381	1.064	0.190	0.096	0.440
	QP	0.577	0.213	0.723	0.168	0.159	0.484
	ARIMA	0.660	0.194	0.385	0.221	0.234	0.413
	BP	2.026	0.819	1.521	0.268	0.243	0.064
	LSTM	0.435	0.311	0.398	0.087	0.059	0.171
	CNN-LSTM-Attention	0.221	0.106	0.238	0.063	0.055	0.040
2 h	LP	1.465	0.404	1.294	0.231	0.115	0.527
	QP	0.901	0.206	0.999	0.194	0.197	0.587
	ARIMA	1.322	0.272	0.641	0.425	0.420	0.787
	BP	2.225	1.145	1.818	0.442	0.371	0.086
	LSTM	0.807	0.341	0.585	0.134	0.104	0.313
	CNN-LSTM-Attention	0.275	0.140	0.346	0.089	0.081	0.043
4 h	LP	1.660	0.582	1.680	0.300	0.189	0.631
	QP	1.526	0.293	1.485	0.248	0.292	0.762
	ARIMA	2.796	0.398	1.052	0.839	0.737	1.493
	BP	3.486	2.742	3.099	1.198	0.964	0.357
	LSTM	1.291	0.390	1.015	0.246	0.213	0.546
	CNN-LSTM-Attention	0.301	0.189	0.437	0.128	0.153	0.056
6 h	LP	1.922	0.695	2.095	0.399	0.261	0.771
	QP	2.020	0.349	2.085	0.312	0.381	0.939
	ARIMA	4.048	0.528	1.513	1.261	1.049	2.218
	BP	8.542	6.564	6.061	2.753	2.256	0.965
	LSTM	1.649	0.519	1.661	0.393	0.337	0.777
	CNN-LSTM-Attention	0.286	0.274	0.471	0.193	0.223	0.060

**Table 6:** Kinematic PPP Accuracy Statistical Results.

Stations	Prediction Data Mean/cm			Post-Processed Products Mean/cm		
	N	E	U	N	E	U
AGGO	0.8	7.3	1.3	−3.3	5.2	0.0
AREG	5.2	8.6	−1.8	7.3	6.6	1.4
AREQ	−0.4	7.9	−3.3	1.1	3.8	0.6
CIBG	7.4	−1.4	−4.9	3.9	−0.2	−1.3
CKSV	−13.9	−3.8	2.7	−1.0	−2.5	2.8
DAV1	13.3	4.2	−3.2	−4.4	0.3	−0.5
GAMG	−10.9	7.3	13.0	−0.9	−2.5	0.7
GRAC	−0.2	9.7	5.4	−0.5	2.6	−2.4
KARR	2.8	0.6	−3.9	5.5	−1.1	−2.4
KMNM	−16.9	−3.7	8.4	−4.2	−1.7	5.3
KOUG	45.8	10.8	9.9	41.0	5.5	8.0
MIZU	−13	7.4	−15.6	−2.0	−2.0	−5.5
ORID	7.9	10.1	−2.5	1.8	−4.2	5.6
P053	−6.7	−1.8	7.8	−0.2	0.5	−0.4
PERT	10.0	1.3	−4.0	6.7	0.8	0.1
RGDG	2.8	8.5	1.3	−3.2	1.7	−0.9
SFDM	−11.4	5.4	8.7	1.7	0.4	2.9
SOD3	10.1	−7.4	−9.9	2.3	−0.8	0.1
STHL	1.4	8.3	4.5	−2.5	1.2	0.9
STR1	6.7	−1.7	−8.4	0.4	0.5	−1.1
TEJA	15.5	16.6	5.4	7.9	9.7	4.2
ULAB	8.4	−7.9	1.3	6.2	−0.5	−2.1
USN7	−14.5	4.5	6.4	0.5	−1.8	−0.6
USUD	−16.1	10.6	3.7	−2.4	−0.9	−1.0
WSRT	16.0	−3.6	10.0	1.8	−2.8	5.9
WTZZ	23.9	−20.8	0.8	2.4	−5.3	3.6
WUH2	−12.1	−3.9	3.9	−1.9	−3.4	0.7
WUTH	11.2	8.4	0.9	0.1	−0.1	−0.5
YEL2	5.9	2.0	5.4	−0.1	0.9	−0.3
YKRO	−29.4	17.2	−25.0	−5.4	4.3	−5.7

**Table 7:** Positioning Accuracy of the Prediction Data.

Stations	N (cm)	E (cm)	U (cm)	Stations	N (cm)	E (cm)	U (cm)
AGGO	2.2	2.2	5.4	RGDG	3.4	12.2	5.8
AREG	2.3	11.4	21.4	SFDM	4.2	10.8	23.5
AREQ	4.3	10.6	18.5	SOD3	2.0	11.3	4.5
CIBG	4.8	2.3	15.3	STHL	9.5	9.1	6.8
CKSV	7.9	13.4	8.0	STR1	10.6	3.1	17.0
DAV1	15.7	26.5	32.2	TEJA	5.3	14.1	11.7
GAMG	9.8	13.7	35.8	ULAB	2.1	10.9	14.1
GRAC	5.2	7.0	11.7	USN7	3.3	10.3	23.0
KARR	4.3	2.5	4.0	USUD	7.8	17.8	20.2
KMNM	8.4	15.9	12.7	WSRT	4.6	15.3	17.2
KOUG	31.3	32.8	106.7	WTZZ	18.1	23.0	4.0
MIZU	5.7	15.4	23.1	WUH2	7.8	9.8	13.0
ORID	10.7	8.1	10.6	WUTH	14.0	13.6	11.2
P053	6.5	5.4	10.2	YEL2	6.8	3.5	10.2
PERT	3.5	2.6	17.2	YKRO	23.7	26.2	34.5

## Data Availability

Both training data and accuracy testing data used in this study are from the post-processing high-precision SCB products provided by IGS WHU. Among them, the experimental data research is publicly available. Available at: https://cddis.nasa.gov/archive/gnss/products (accessed on 25 June 2025).
